# A controlled study comparing salivary osmolality, caries experience and caries risk in patients with cerebral palsy

**DOI:** 10.4317/medoral.22135

**Published:** 2018-02-25

**Authors:** Luciana-Angélica Ruiz, Michele-Baffi Diniz, Juan-Pablo Loyola-Rodriguez, Carolina-Hartung Habibe, Claudia-Cinelli Garrubbo, Maria-Teresa-Botti-Rodrigues Santos

**Affiliations:** 1 Associação de Assistência à Criança Deficiente - AACD, São Paulo, SP, Brazil; 2 Postgraduate Program in Dentistry, Cruzeiro do Sul University, São Paulo, SP, Brazil; 3 Escuela Superior de Odontología, Universidad Autónoma de Guerrero, Acapulco, México; 4 School of Dentistry, Centro Universitário de Volta Redonda – UniFOA, Volta Redonda, RJ, Brazil

## Abstract

**Background:**

Cerebral palsy (CP) is a permanent neurological disorder accompanied by secondary musculoskeletal masticatory disorder, with repercussion on chewing and deglutition functions. In these conditions, the liquids ingestion is compromised resulting in salivary osmolality alteration. The objective of this study was to compare salivary osmolality, caries experience and caries risk between normoreactive individuals and patients with CP.

**Material and Methods:**

The participants were 4-20 years old: 52 patients with CP treated at a reference rehabilitation centre (study group, SG), and 52 normoreactive individuals (control group, CG). Saliva was collected for five minutes using cotton rolls. Following centrifugation, salivary osmolality was determined by freezing point depression osmometry. Evaluations included caries experience (DMFT index), and caries risk based on a caries-risk assessment tool (CAT). Descriptive and inferential statistics (Chi square and Student t tests) were used to compare the groups. Receiver operating characteristic (ROC) analyses were performed and the area under the ROC curve (Az) was calculated. The level of significance was set at 5%.

**Results:**

The groups were homogeneous for sex (*p*=0.843) and age (*p*=0.128). In the SG, spastic type CP was the most prevalent (80.8%), and patients showed significantly higher salivary osmolality values compared with the CG (*p*<0.001). No significant differences in caries experience (*p*=0.159) or caries risk (*p*=0.297) were observed. ROC curve analysis determined a salivary osmolality cutoff point of >74 for the SG and >54 for the CG in the presence of dental caries. A significant correlation was verified between salivary osmolality and the DMFT index for the SG (*p*≤0.05).

**Conclusions:**

Although patients with CP showed higher salivary osmolality values, higher caries experience and caries risk were not observed compared with normoreactive individuals.

** Key words:**Cerebral palsy, osmolar concentration, dental caries, saliva.

## Introduction

Cerebral palsy (CP) describes a group of chronic disorders that involve movement and posture development often accompanied by epilepsy, secondary musculoskeletal problems and disturbances of sensation, perception, cognition, communication and behaviour. It is the most common cause of severe physical disability in childhood (1), with an estimated prevalence of 2.4 per 1000 children (2).

Individuals with CP present a high prevalence of dental caries (3-7), oral motor dysfunction (8), shorter mastication endurance time (9), biting reflexes (10), regular use of sugary anticonvulsant drugs (11), intellectual disability (12) and worse quality of life and continual burden on their caregivers (4) that can further influence oral hygiene (8). Moreover, a reduction in salivary flow and pH, compromise of the buffer capacity (13), changes in enzyme activity and sialic acid concentration (14) have been reported.

Individuals with CP also show increased salivary osmolality and total proteins (15) associated with dehydration (16). Decreased levels of hydration can result in diminished salivary output, which can compromise the protective function exerted by saliva and increase the risk of oral diseases (16). It is worth noting that studies on salivary osmolality, caries experience and caries risk in individuals with CP show no comparative results with groups of normoreactive individuals (6,15,17).

Given the above, the purpose of this study was to compare salivary osmolality, caries experience and caries risk between normoreactive individuals and those with CP. The hypothesis tested was that since individuals with CP present higher salivary osmolality, they also present higher caries experience and caries risk.

## Material and Methods

- Participants

The project was approved by the Research Ethics Committee of the Association for the Care of Disabled Children (AACD), under protocol no. 260.255 as registered on Plataforma Brasil. The parents or guardians of the participants signed a term of free, informed consent authorising their participation in the study.

The study selected 104 individuals between 4 and 20 years of age, of both sexes, all from the State of São Paulo, Brazil. The study group (SG) consisted of 52 individuals with cerebral palsy (CP) treated at a reference rehabilitation centre, with periodic returns every 3-6 months, and the control group (CG) consisted of 52 normoreactive individuals who also periodically received dental care. The control group was composed by individuals who sought dental treatment in Pediatric Dentistry and Integrated Clinic departments in a private University in São Paulo, Brazil. One of the co-authors was responsible to select those individuals during data collection, and they should not be taken any kind of drugs.

Only individuals with a medical diagnosis of CP were included in the SG. The exclusion criteria included individuals with who had taken drugs that could interfere with salivary secretion such as anticholinergics, neuroleptics or benzodiazepines, and individuals who presented diagnoses concomitant with CP. In the present study the CP participants were classified according to the movement disorder as spastic including the clinical parameters tetraparesis (involvement of all four limbs), diparesis (the legs are more involvement than arms) and hemiparesis (ipsilateral arm and leg); dyskinetic, and mixed type (association between spastic and dyskinetic). This information was assessed by examining patient medical records.

- Saliva collection and assessment

The participants were asked not to eat, drink or brush their teeth in the 2-h period prior to the collection of whole saliva. Saliva was collected using an absorbent cotton roll (18) (Salivette®, Sarsted, Numbrecht, Germany) placed on the mouth floor for 5 min. Saliva collection was conducted in a ventilated, well-lit room, with the patient sitting in an upright position. The saliva samples were frozen at -20°C immediately following collection.

After thawing at room temperature for 1 h, the Salivette® cotton rolls were centrifuged at 5,000 rpm at 4°C for 5 min (Hettich, Universal 320R centrifuge, Tuttlingen, Germany). Salivary osmolality was measured with a freezing point depression osmometer (Model Vapro Vapor Pressure Osmometer 5600, New Instrument, Washington, DC, USA) (19).

- Caries experience

A single calibrated examiner (weighted Kappa=0.91) performed the oral examinations, with the help of an assistant to record the data. Before the clinical examination, professional tooth cleaning was performed on each patient. The patient’s teeth were assessed under a reflector light using a dental mirror and a probe after drying with a 3-in-1 syringe. The World Health Organization criteria were used to record dental caries experience using the decay, missing and filled indices, dmft and DMFT, for primary and permanent dentition, respectively. In children with mixed dentition, dmft and DMFT were recorded together. No radiographic examination was performed. The caries experience score was classified according to severity: very low (0.0 to 1.1), low (1.2 to 2.6), moderate (2.7 to 4.4), high (4.5 to 6.5) and very high (6.6 and above) (20).

The risk of caries was assessed using the Caries-risk Assessment Tool (CAT) (21) recommended by the American Academy of Pediatric Dentistry (AAPD). The biological and protective factors and clinical features of each individual were assessed.

- Statistical analysis 

The data collected were tabulated and analysed by MedCalc for Windows software using descriptive and inferential statistics (Chi square and Student t tests) to compare the groups.

For both groups, the individuals were separated into no caries experience (=0) and caries experience (≥1). Receiver operating characteristic (ROC) analyses were performed and the area under the ROC curve (Az) was calculated. The best cutoff point for salivary osmolality for each group was determined following the ROC ana-lyses, considering the sum of specificity and sensitivity.

Spearman’s correlation coefficient was calculated for each group, considering the salivary osmolality and caries experience data. The level of significance was set at 5% (*p*<0.05).

## Results

The 52 participants with CP who composed the SG were aged between 4 and 20 years old (10.2 ± 4.7), 23 (44.2%) were female and 29 (55.8%) were male. The CG was composed of 52 normoreactive individuals aged between 4 and 20 years old (9.6 ± 4.2), 22 (42.3%) were female and 30 (57.7%) were male. The groups were homogeneous for sex (*p*=0.843) and age (*p*=0.128).

Regarding the distribution of movement disorder in the SG, 42 (80.8%) were spastic type CP, 7 (13.5%) were dyskinetic, and 3 (5.7%) of mixed type. Among those with spasticity, 16 (38.1%) had tetraparesis, 17 (40.5%) had diparesis and 9 (21.4%) had hemiparesis.

Calculation of the power of the study for the sample was 99.4%, considering the salivary osmolality values of the groups, with alpha=5% (OpenEpi).

The mean and standard deviation of salivary osmolality and caries experience (DMFT index) are presented in  Table 1. The SG presented significantly higher salivary osmolality values compared with the CG. No statistically significant differences in caries experience were observed between the groups.

**Table 1:**
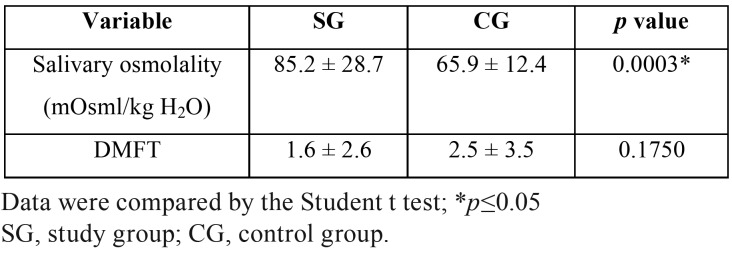
Means and standard deviations for salivary osmolality and the DMFT index for the groups assessed.

For individuals with caries experience (DMFT ≥1), 25 (48.1%) were from the SG (3.4 ± 2.8) and 27 (51.9%) from the CG (4.8 ± 3.5); no statistically significant difference was verified between the groups (*p*=0.264). Salivary osmolality values for individuals with caries experience (DMFT ≥1) differed significantly between the groups (*p*<0.001), with the SG showing higher values (77.7 ± 24.3) compared with the CG (68.4 ± 11.6).

Comparison of the results obtained for the severity of the DMFT index showed no statistically significant difference between the SG and CG (Chi square test, *p*=0.297). Thirty-two (61.6%) participants were classified as “very low” in the SG and 29 (55.7%) in the CG; 11 (21.1%) were classified as “low” in the SG and 8 (15.4%) in the CG; 3 (5.8%) were classified as “moderate” in the SG and 3 (5.8%) in the CG; none were classified as “high” in the SG and 4 (7.7%) in the CG; and 6 (11.5%) were classified as “very high” in the SG and 8 (15.4%) in the CG.

Regarding caries risk, the SG and CG showed no significant differences (Chi square test, *p*=0.467). In the SG, 45 (86.5%) participants were assessed as at low risk, 4 (7.7%) at high and 3 (5.8%) at moderate risk of developing dental caries, while in the CG, 41 (78.8%) participants were assessed as at low risk, 8 (15.4%) at high and 3 (5.8%) at moderate risk.

**Figure 1:**
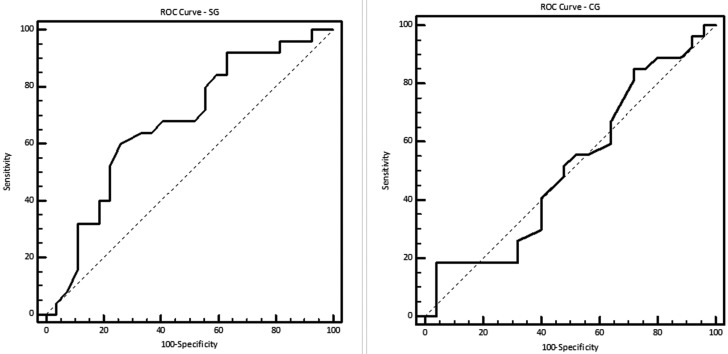
ROC curve analysis for the study (SG) and control groups (CG).

ROC curve analysis for the SG showed a cutoff point of >74 for salivary osmolality in the presence of dental caries, with an area under the curve of 0.674 (95% confidence interval 0.526-0.822), a sensitivity of 60.0% and a specificity of 74.1%. For the CG, ROC curve analysis showed a cutoff point of >54 for salivary osmolality in the presence of dental caries, with an area under the curve of 0.513 (95%CI 0.353-0.674), a sensitivity of 18.5% and a specificity of 96.0% (Fig. 1).

The Spearman correlation coefficient between salivary osmolality and the DMFT index is shown in  Table 2. Only the SG showed a significant association between these variables (*p*=0.05).

**Table 2:**
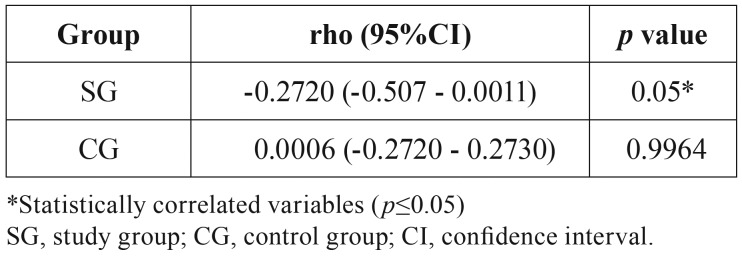
Spearman correlation coefficient (rho) between salivary osmolality and the DMFT index for the groups assessed.

## Discussion

To our knowledge, this is the first study that compares salivary osmolality, dental caries experience and caries risk in CP and normoreactive individuals. The hypothesis of the study was that individuals with CP would present higher caries experience and higher caries risk, since the higher salivary osmolality values described in the literature are considered to be an additional risk factor for oral disease in these individuals (6,15-17). However, this hypothesis was not confirmed when the results obtained from the CP (SG) and normoreactive participants (CG) were compared.

Several studies indicate that individuals with CP have increased risk of oral diseases, such as dental caries (3-7). However, in this investigation, no difference in caries experience was verified between the SG and CG. This could be explained by the effective control of caries risk factors in individuals with CP, through a process that involves growing caregiver knowledge concerning oral hygiene habits, the use of open mouth devices to facilitate tooth brushing and diet control. The fact that the patients were treated in a rehabilitation centre providing constant preventive care could also be a factor in the low DMFT index obtained compared with previous studies (4,8).

In previous studies, higher values for salivary osmolality have been reported for individuals with CP (6,15,16). The results obtained herein showed significant greater values for the SG compared with the CG and a significant correlation between salivary osmolality and caries experience for the SG. Among individuals with CP who cannot sustain lip closure, the liquid diet intake usually flows out of the mouth, resulting in a lack of negative pressure for swallowing, which can cause choking. Consequently, for these individuals, water intake is generally below required levels, since they tend to consume small volumes of liquids (22).

Higher salivary osmolality values imply greater salivary viscosity due to changes in saliva constitution, reducing the potential outflow of saliva (23), together with its clearance capacity, which facilitates bacterial assemblage in the acquired film and biofilme (24). The presence of this chronic saliva status can lead to salivary gland dysfunction (25), which would explain the higher salivary osmolality values observed for the SG. The cutoff point determined by the ROC curve analysis for salivary osmolality of the SG (> 74) was similar to previous results published by our group (6).

For the CG, the lower salivary osmolality values obtained could be related to the presence of competent oral motor skills, which means that this group is subject to known etiological factors for caries, with little or no impact due to salivary osmolality. Thus, it can be inferred that the salivary osmolality did not represent an additional risk factor for hydrated normoreactive individuals, since dental caries experience were similar for both groups. To our knowledge, there is no previous study in the literature evaluating salivary osmolality and caries risk in normoreactive individuals. In this context, it is necessary future investigations comparing normoreactive individuals with different dental caries experience and salivary osmolality to determine its influence on caries risk.

According to the Caries-risk Assessment Tool recognised by the American Academy of Pediatric Dentistry, both groups in this study showed low risk for caries (21), and low severity for caries experience (20). However, it is important to note that these groups differ regarding oral motor behaviour, since neurological injury leads to musculoskeletal problems in the mastication and swallowing muscles (1), interfering in oral motor skills, in diet consistency and in liquid intake, further strengthening the hypothesis that those with neurological injuries show different behaviour for oral diseases compared with normoreactive individuals. It is important for the pediatric dentists to know about this issue (26), recognize the signs or symptoms and provide dental treatment to children with neurological damages.

Based on these findings, it seems reasonable to assume that a preventive approach is fundamental to control the caries experience of individuals with CP. This requires greater focus on specific issues, including detailed advice concerning sealant application, fluoride therapy, the use of alcohol-free 0.12% chlorhexidine digluconate mouth rinse, tooth brushes adapted to facilitate handling by individuals with CP, the use of dental floss devices, single-tuft toothbrushes for use on occlusal surfaces, and individual mouth opening. It is also important to emphasise the need for dental consultations scheduled at short intervals to ensure the best professional care, including adequate follow-up of erupting teeth on an individual basis.

It is important that when the dentist or dental surgeon observe the presence of viscous saliva in children with or without CP, that they orient the caregivers, parents or guardians to increase the provision of liquid intake, preferably water, maintaining the electrolyte balance of the saliva so that it can fulfil its protective role in the oral cavity.

## Conclusions

Analysis of the results clearly indicates that although patients with cerebral palsy show higher salivary osmolality, they do not present higher caries experience or higher caries risk than normoreactive individuals.
